# syn-tasiRnas targeting the coat protein of potato virus Y confer antiviral resistance in *Nicotiana benthamiana*

**DOI:** 10.1080/15592324.2024.2358270

**Published:** 2024-05-26

**Authors:** Xingyue Zhao, Qian Gao, Haijuan Wang, Jianying Yue, Derong An, Bin Li, Fangfang Yan, Simon-Mateo Carmen, Yuanzheng Zhao, Hongyou Zhou, Mingmin Zhao

**Affiliations:** a College of Horticulture and Plant Protection, Inner Mongolia Agricultural University, Hohhot, China; b College of Plant Protection, Northwest A&F University, Yangling, China; c Department of Tabacco Production, Sichuan Province Company of Tobacco Corporation in China, Chengdu, China; d Panzhihua City company of Sichuan province company of Tobacco Corporation in China, Panzhihua city, Sichuan provience, China; e Centro Nacional de Biotecnologia, CSIC, Madrid, Spain; f Department of Plant Protection, Inner Mongolia Academy of Agricultural and Animal Husbandry Sciences, Hohhot, China; g Department of Plant protection, Key Laboratory of the Development and Resource Utilization of Biological Pesticide in Inner Mongolia, Hohhot, China

**Keywords:** Potato, potato virus Y, trans-acting small interfering RNA, synthetic sirnas

## Abstract

Trans-acting small interfering RNAs (tasiRNAs) are 21-nt phased (phased siRNAs) resulting from successive DCL-catalyzed processing from the end of a double-stranded RNA substrate originating from the RDR of an AGO-catalyzed cleaved RNA at a micro RNA target site. Plant tasiRNAs have been synthesized to produce synthetic tasiRNAs (syn-tasiRNAs) targeting viral RNAs that confer viral resistance. In this study, we engineered syn-tasiRNAs to target potato virus Y (PVY) infection by replacing five native siRNAs of TAS1c with 210-bp fragments from the coat protein (CP) region of the PVY genome. The results showed that the transient expression of syn-tasiR-CPpvy2 in Nicotiana benthamiana (N. benthamiana) plants conferred antiviral resistance, supported by the absence of PVY infection symptoms and viral accumulation. This indicated that syn-tasiR-CPpvy2 successfully targeted and silenced the PVY CP gene, effectively inhibiting viral infection. syn-tasiR-CPpvy1 displayed attenuated symptoms and decreased viral accumulation in these plants However, severe symptoms of PVY infection and a similar amount of viral accumulation as the control were observed in plants expressing syn-tasiR-CPpvy3. syn-tasiR-CPpvy/pvx, which targets both PVY and potato virus X (PVX), was engineered using a single precursor. After the transient expression of syn-tasiR-CPpvy/pvx3 and syn-tasiR-CPpvy/pvx5 in N. benthamiana, the plants were resistant to both PVY and PVX. These results suggested that engineered syn-tasiRNAs could not only specifically induce antiviral resistance against one target virus but could also be designed for multi-targeted silencing of different viruses, thereby preventing complex virus infection in plants.

## Introduction

Gene silencing refers to gene regulation at the genetic level and can be divided into transcriptional gene silencing (TGS) and post-TGS (PTGS).^1^ In particular, RNA interference (RNAi) is an important PTGS in eukaryotic organisms that is triggered by the introduction of double-stranded RNA (dsRNA), leading to gene silencing in a sequence-specific manner.^2^ Long dsRNAs are cleaved by an RNase III family member, Dicer, into 21–24-nucleotide (nt) fragments with 5′ phosphorylated ends and 2-nt unpaired and unphosphorylated 3′ ends. These small dsRNAs are called small interfering RNAs (siRNAs). Each siRNA duplex is formed by a guide strand and passenger strand, which are catalyzed by the endonuclease Argonaute (AGO) to unwound the siRNA duplex. Once unwound, the guide strand is incorporated into the RNAi specificity complex (RISC), whereas the passenger strand is released. The guide strand carried by the RISC specifically matches the complementary sequence of the mRNA, leading to the cleavage of the target mRNA.^3^

Since its discovery, RNAi has become an important tool for gene silencing and antiviral resistance in plants.^4–6^ Antiviral resistance can be experimentally induced by the exogenous introduction of dsRNA or constructs that express short hairpin RNA.^7–10^ Moreover, technology based on artificial microRNAs (amiRNAs) exploits RNAi mechanisms to silence endogenous genes or pathogens and has been successfully employed to induce resistance against different eukaryotic viruses.^11–15^

Trans-acting siRNAs (tasiRNAs) are phased siRNAs (21–22-nt long) with trans-acting regulatory functions.^16,17^ TasiRNAs have also been identified in various plant species, especially in *Arabidopsis thaliana (A. thaliana)* and *Solanaceae* and *Leguminosae*. TasiRNAs are processed by the microRNA (miRNA)-mediated cleavage of tasiRNA precursors (TAS) gene transcripts are subsequently stabilized by the suppressor of gene silencing 3 (SGS3) and converted to double-stranded RNA (dsRNA) by the action of RNA-dependent RNA polymerase 6 (RDR6).^18^ In *A. thaliana*, the formation of tasiRNA is mediated by miRNA-directed non-protein-encoding TAS genes under the action of Pol II to form pri-miRNA with a stem-loop structure via base pairing,^19^ then generating an miRNA:miRNA*double-stranded structure with the help of SGS3 and RDR6.^20^ According to different miRNA initiation cleavage sites, double-stranded molecules are cleaved into short siRNAs starting at the end of the miRNA cleavage site in units of 21nt^21^ or 22nt.^22^ Four families of genes encoding TAS, comprising eight different loci, have been identified in the *A. thaliana* genome.^23^ The TAS3 family generates tasiRNAs via a two-hit mechanism triggered by miR390 loaded into AGO7. The primary transcripts of the TAS1/TAS2 family are targeted by a single hit of the 22-nt-long version of miR173 in *A. thaliana* and other closely related species.^24,25^ The three target genes of miR173(At2g27400, At1g50055, and At2g39675) encode tasiRNAs and are, therefore, named TAS1a, TAS1b, and TAS1c, respectively.^26,27^ In particular, the TAS1c transcript was engineered to process artificially trans-acting siRNAs to confer consistent and effective gene silencing.^28,29^ Replacing the endogenous siRNAs encoded in the TAS1c gene with sequences from the FAD2 gene silenced FAD2 activity to levels comparable to the *fad2–1* null allele in nearly all transgenic events. miR173 was specifically required to trigger the production of siRNAs from the TAS1c locus, which was demonstrated by exchanging the endogenous miR173 target sequence in TAS1c with the miR167 target sequence, leading to variable, inefficient silencing of FAD2.^28^ miR173-mediated tasiRNA production can be applied to exogenous plants, such as *N. benthamiana*, and has been engineered to silence endogenous genes with single or multiple targets, including FAD2,^28^ PDS,^30^ CH42,^31^ FT, or Try/CPC/ETC2.^32^ Synthetic tasiRNAs (syn-tasiRNAs) were engineered by artificially replacing the TAS1c sequence with the complementary sequences of target genes in plants so that the constructed syn-tasiRNA could specifically inhibit the expression of target sequences. In 2015, Singh et al. designed a syn-tasiRNA targeting the AC2 and AC4 genes of the tomato leaf curl New Delhi virus. The expression of syn-tasiRNA in both tobacco and tomato plants resulted in the abundant production of specific siRNAs targeting and silencing the viral genes, leading to enhanced resistance in the plants.^33^ To test the antiviral effect against Plum pox virus (PPV), Zhao et al. modified the TAS1c transcript by replacing two 210-bp fragments from the coat protein (CP) and 3′ non-coding regions of the PPV genome.^22^ Further, Baykal et al. found that the formation of an integral miR173-TAS1a system from a pre-miR173 and TAS1a co-conjugated promoter was more effective than gene silencing by two independent miR173 and TAS locus co-expression systems, achieving gene silencing without lethality.^34^ Carbonell et al. found that syn-tasiRNAs could be effectively used to control PSTVd virus in plant.^35^ They also designed a construct to simultaneously express a combination of syn-tasiRNAs targeting multiple genes of the tomato spotted wilt virus. Syn-tasiRNAs of multiple target genes can combine the disease resistance of a single tasiRNA to minimize the possibility of mutating all targets simultaneously and increase the effect of syn-tasiRNA silencing.^36^ By targeting different positions on the potato virus Y (PVY) CP gene, Wu et al. engineered three plant syn-tasiRNAs based on the TAS3a locus and transferred them into tobacco. They found that all transgenic plants containing syn-tasiRNAs were virus resistant.^37^ Syn-tasiRNAs mixed with amiRNAs targeting the conserved regions of different strains of the cucumber green mottle mosaic virus (CGMMV) genome successfully blocked CGMMV accumulation in cucumber.^38^

PVY is a plant virus of the genus *Potyvirus* that naturally infects potatoes, but it is also able to infect a wide range of *Solanum* plants.^39^ Potyviruses contain a positive-sense RNA genome of approximately 10 kb, encoding a genome-length polyprotein and a frameshift product derived from RNA polymerase slippage, which is processed by three viral proteases.^40,41^ Potato virus X (PVX) is an important virus that infects potatoes and usually causes mosaic symptoms and mixed infection with PVY in the field, which often leads to the devastation of potatoes.^42–44^ PVX is a member of the genus Potexvirus, a group of plant viruses with a positive-stranded RNA genome (~6.4 kb).^45^ In this study, we engineered syn-tasiRNAs targeting the CP gene in PVY. Multiple syn-taisRNAs targeting both the PVY and PVX CP were designed in a single expression construct. The effects of PVY and PVX infection on the expression of different virus-specific syn-tasiRNAs in *N. benthamiana* were assessed.

## Results

### The design of syn-tasiRnas targeting the CP gene of PVY

**Figure 1.**
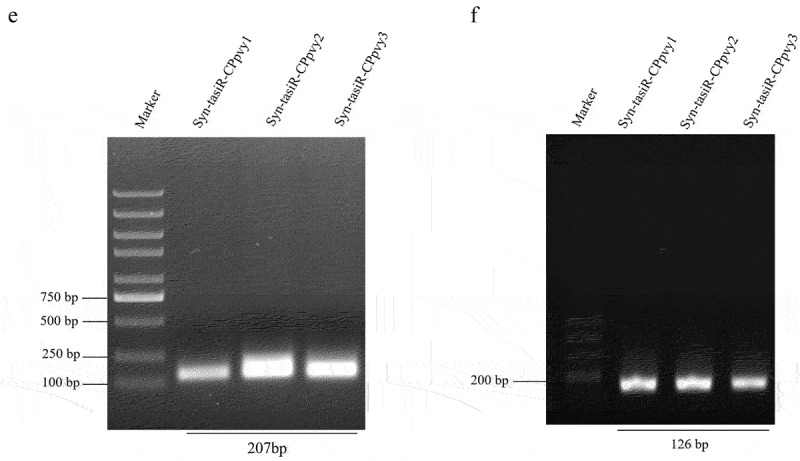
(Continued).

In the TAS1c gene sequence of *A. thaliana*, miR173 identified the 3′ end of the cleavage site sequentially generating six tasiRNAs named 3′D1(+), 3′D2(+), 3′D3(+), 3′D4(+), 3′D5(+), and 3′D6(+) (Figure 1A). Among them, 3′D3(+) and 3′D4(+) accumulated more, corresponding to tasiRNAs named siR255 and siR860, respectively. 3′D6(+) was distantly located from the start site of tasiRNA production, accumulated less, and corresponded to the tasiRNA named siR619. Based on the biological characteristics of tasiRNA production from TAS1c, we cloned a modified TAS1c sequence from *A. thaliana* including the recognition site for miR173, into the plant binary vector pMDC32 (Figure 1B). The siRNA production sequences were then replaced with selected CP regions of the PVY sequences (Figure 1C). In this construct, six tasiRNAs derived from the viral sequences were expected to be sequentially produced after the miR173-specific cleavage site (Figure 1D). The sequences of syn-tasiR-CPpvy1, syn-tasiR-CPpvy2, and syn-tasiR-CPpvy3 are presented in Figure S6. The syn-tasiR-CPpvy2 (433–559 nt) sequence is shown in Figure 1C and Figure 1D.
**Figure 1.**
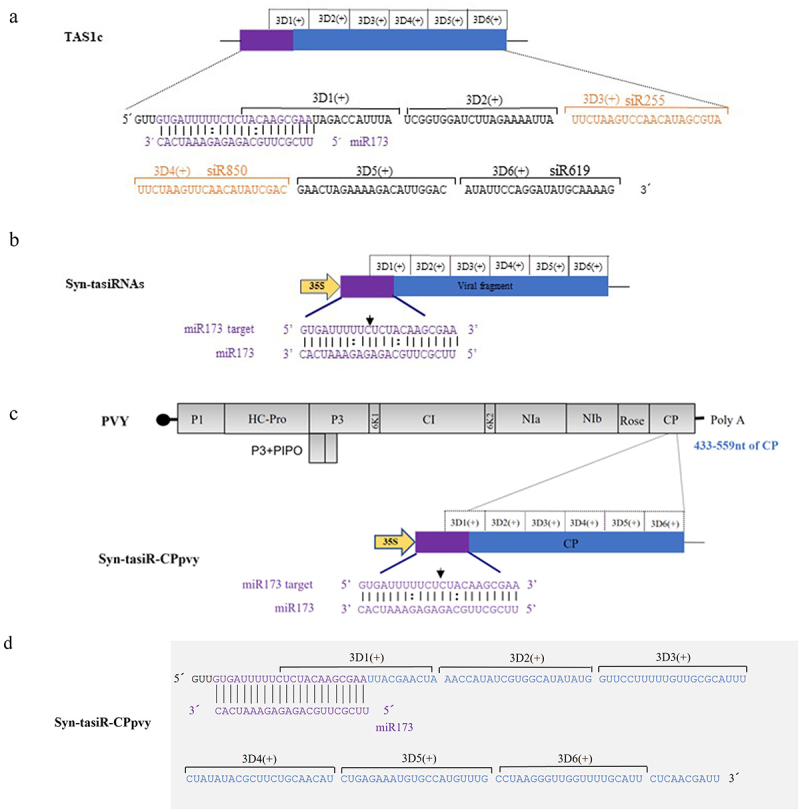
The design and construction of Syn-tasiR-CPpvy. (a) The construction of the TAS1c gene and tasiRnas sequences; (b) the construction of syn-tasiRnas containing viral sequences; (c) Schematic of the PVY genome and the design of Syn-tasiR-CPpvy based on CP of PVY; (d) the sequence of Syn-tasiR-CPpvy; (e) the PCR fragments of syn-tasiR-CPpvy; and (f) PCR amplification of specific bands from pMDC32-syn-tasiR-CPpvy.

DNA fragments of the viral CP gene were amplified and subjected to gel electrophoresis. The 207-bp fragments shown in Figure 1D were then ligated into the pMD19-T vector for sequencing validation (Figure S1). Using the BP reaction of the Gateway recombination system, three fragments were cloned into pDONR207 to obtain pDONR207-syn-tasiR-CPpvy1, pDONR207-syn-tasiR-CPpvy2, and pDONR207-syn-tasiR-CPpvy3. The fragment in pDONR207 was cloned into the binary vector pMDC32 using the LR reaction of the Gateway recombination system to obtain pMDC-syn-tasiR-CPpvy1 (syn-tasiR-CPpvy1), pMDC-syn-tasiR-CPpvy2 (syn-tasiR-CPpvy2), and pMDC-syn-tasiR-CPpvy3 (syn-tasiR-CPpvy1) and verified by *Age-HF* digestion resulting in expected enzymatic segments of 6550/3670 bp, 6553/3667 bp, and 6553/3670 bp, respectively (Figure S2 and S3). Further, the polymerase chain reaction (PCR) amplification of specific bands of syn-tasiR-CPpvy1, syn-tasiR-CPpvy2, and syn-tasiR-CPpvy3 resulted in specific amplifications, each with a specific band of 126 bp (Figure 1F). This indicated the successful construction of a recombinant dual-expression vector for syn-tasiR-CPpvy.

### Transient expression of syn-tasiRnas targeting the PVY CP gene in N. benthamiana confers antiviral resistance

**Figure 2.**
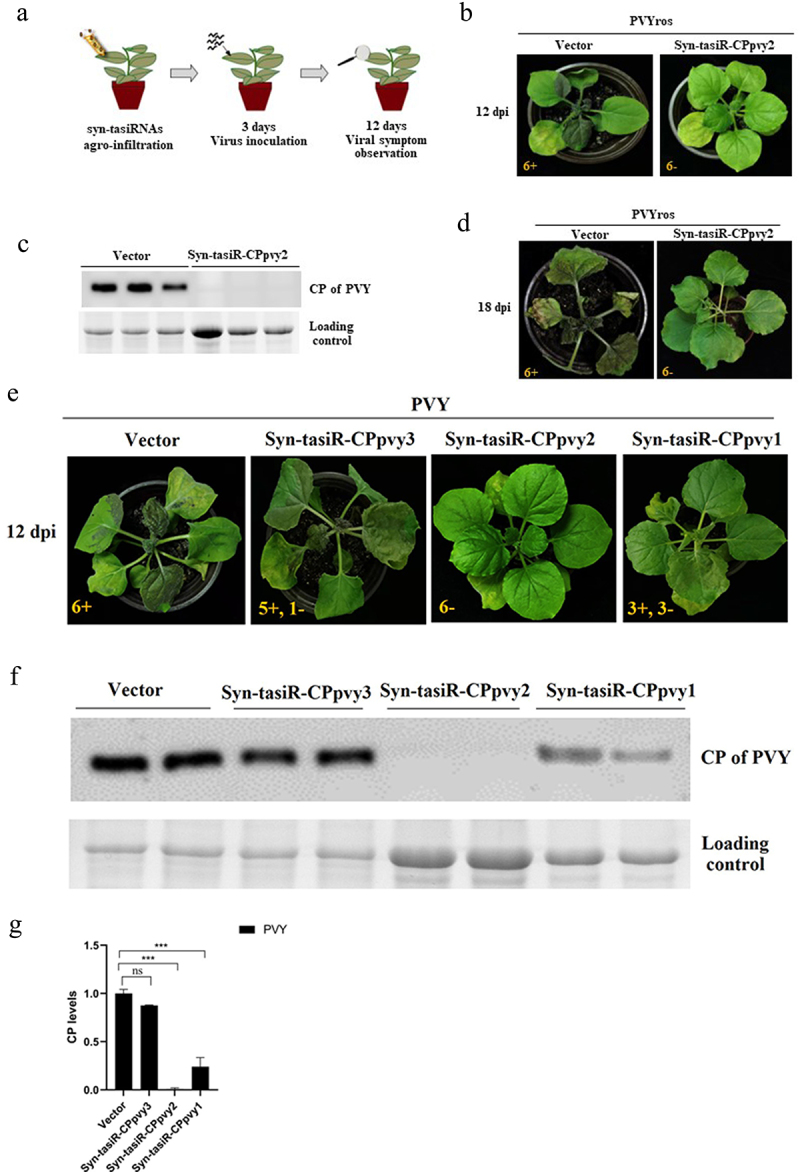
The antiviral effect of Syn-tasiR-CPpvy on PVY infection. (a) The procedure of syn-tasiRnas agroinfiltration for the antiviral test in *N. benthamiana*; (b) the symptoms of PVY in *N. benthamiana* (at 12 dpi) expressing Syn-tasiR-CPpvy2; (c) Western blotting-detected viral accumulation of PVY in plants infiltrated with syn-tasiR-CPpvy2. The protein band of Rubisco was used as a loading control. (d) The symptoms of PVY in *N. benthamiana* (at 18 dpi) expressing Syn-tasiR-CPpvy2; (e) the symptoms of PVY in *N. benthamiana* (at 12 dpi) expressing Syn-tasiR-CPpvy1, Syn-tasiR-CPpvy2, and Syn-tasiR-CPpvy3; (f) Western blotting-detect viral accumulation of PVY in plants infiltrated with syn-tasiR-CPpvy1, syn-tasiR-CPpvy2, and syn-tasiR-CPpvy3. The protein band of Rubisco was used as a loading control. The number in each photo represents the number of tested plants. The “+” sign represents that the plant shows typical symptoms of viral infection; the “–” sign represents that the plant shows no symptoms of viral infection. (g) The CP signal was quantified using Quantity One. The statistical analysis and P-values analyzed by ANOVA.

The expression vectors pMDC32-syn-tasiR-CPpvy and pMDC2-miR173 were transformed into *A. tumefaciens* C58C1 and co-infiltrated into the leaves of *N. benthamiana*. Three days later, the viral extract of PVYros was inoculated into the infiltrated leaves. The antiviral effect was assessed by observing viral infection symptoms and viral accumulation detected by western blotting (Figure 2a). The results showed that there were no viral infection symptoms in any of the plants transiently expressing syn-tasiR-CPpvy2, whereas all plants infiltrated with the empty vector pMDC32 showed obvious PVYros infection symptoms on leaves displaying red necrosis at 12 dpi (Figure 2b). The samples were subjected to the detection of viral accumulation by western blot analysis using a specific antibody against the PVY CP protein. The results showed that No bands were detected in plants transiently expressing syn-tasiR-CPpvy2, whereas a large CP-specific band was detected in the leaves of plants expressing the empty vector (Figure 2c). Plants infiltrated with syn-tasiR-CPpvy2 were free of viral infection symptoms at 18 dpi. This indicates that the transient expression of syn-tasiR-CPpvy2 may confer antiviral resistance against PVY in *N. benthamiana*.

**Figure 4.**
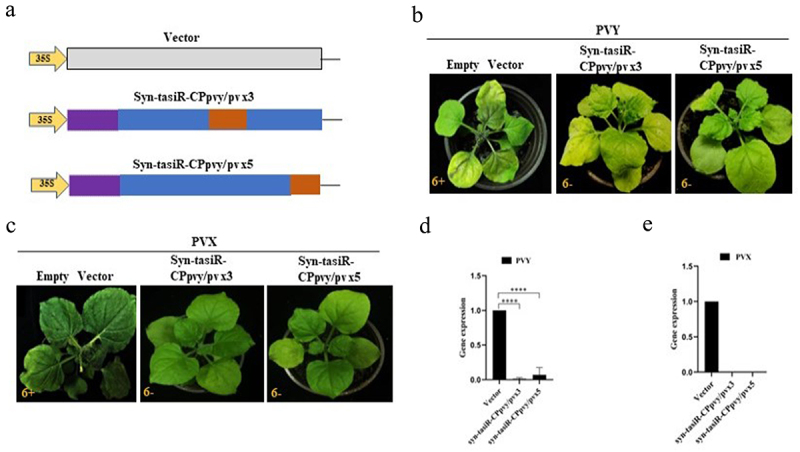
The antiviral effect of syn-tasiRnas on PVY and PVX infection. (a) A schematic diagram showing the structures of the expression vectors pMDC32-syn-tasiR-CPpvy/pvx3 and pMDC32-syn-tasiR-CPpvy/pvx5 and the empty vector pMDC32; (b) the symptoms of PVY in *N. benthamiana* (at 12 dpi) expressing syn-tasiR-CPpvy/pvx3 and syn-tasiR-CPpvy/pvx5; (c) the symptoms of PVX in *N. benthamiana* (at 12 dpi) expressing syn-tasiR-CPpvy/pvx3 and syn-tasiR-CPpvy/pvx5; (d) Viral accumulation of PVY or PVX was detected by RT-qPCR in these plants corresponding to each virus-infected plant in B or C.

Further, we performed transient expression assays to test the antiviral effects of syn-tasiR-CPpvy1, syn-tasiR-CPpvy2, and syn-tasiR-CPpvy3 on PVYros. None of the results indicated that all plants expressing syn-tasiR-CPpvy2 exhibited symptoms of viral infection. Half of the plants expressing syn-tasiR-CPpvy1 showed symptoms of viral infection and others did not. Five of six plants expressing syn-tasiR-CPpvy3 showed symptoms of viral infection. Plants infiltrated with the empty vector exhibited severe symptoms of viral infection (Figure 2e). Correspondingly, a large amount of viral accumulation was detected by western blot analysis in plants expressing syn-tasiR-CPpvy3, with no significant difference compared with the empty vector control. Plants expressing syn-tasiR-CPpvy1 showed a significant decrease in viral accumulation compared with that in the control (Figure 2f). Signal quantification and statistical analysis showed that the changes in viral protein amount were significant, i.e., a significant reduction in CP accumulation was detected in plants expressing syn-tasiR-CPpvy1 (*p* = 0.0003) or syn-tasiR-CPpvy2 (*p* = 0.0001) compared with that in the control (Figure 4g). This indicates that the target sequence may be important for syn-tRNA-mediated antiviral resistance.

### Design of syn-tasiRnas targeting both PVY and PVX

**Figure 3.**
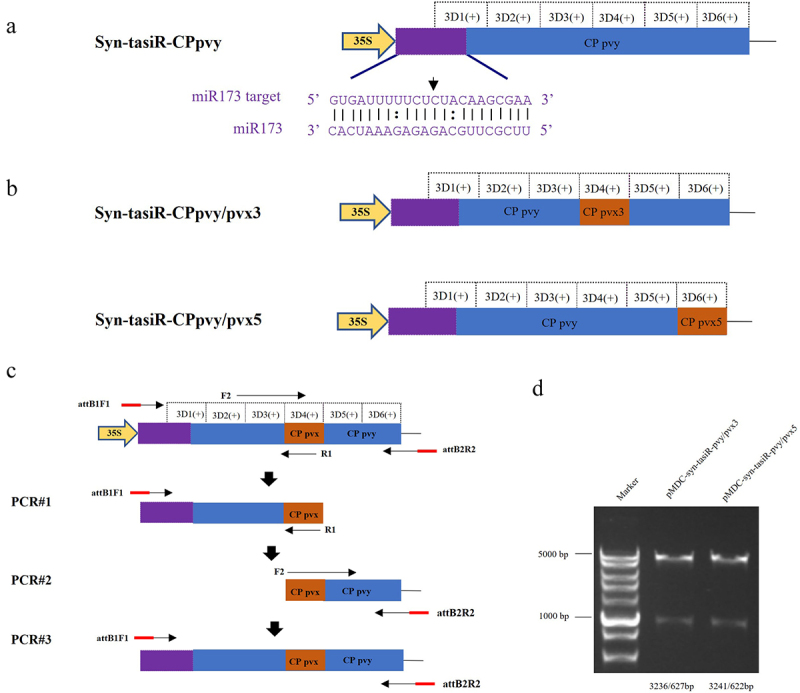
The design of syn-tasiRnas targeting both PVY and PVX. (A) The design of the syn-tasiR-CPpvy expression vector targeting the CP gene of PVY; (B) Based on the syn-tasiR-CPpvy expression vector, the siRNA sequence targeted different positions of the PVX CP gene to create different syn-tasiR-CPpvy/pvx expression vectors. (C) The schematic model of overlap PCR for cloning the constructs of syn-tasiRnas targeting PVY and PVX; (D) the digestion of plasmids using the restriction enzyme *Pst I* to confirm the expression vectors of pMDC32-syn-tasiR-CPpvy/pvx.

To verify whether the same expression vector carrying different syn-tasiRNAs could simultaneously target two types of viruses, we constructed expression vectors containing both syn-tasiRNAs targeting PVY and syn-tasiRNA targeting PVX. One syn-tasiRNA vector was expected to produce five syn-tasiRNAs targeting PVY and one syn-tasiRNA targeting PVX (Figure 3a). Using pMDC32-syn-tasiR-CPpvy2 as a vector background, we replaced the positions of these syn-tasiRNAs with specific PVX targets by PCR amplification to produce pMDC-syn-tasiR-CPpvy/pvx (Figure 3b). Overlap PCR was used to design a pair of primers, using the syn-tasiR-CPpvy2 sequence as a template. The upstream primer was attB1F1 and the downstream primer was R1 containing the PVX-specific fragment, thereby introducing CPpvx into the 3′D4(+) position of R1. A second pair of primers was designed. The upstream primer was F2 (containing the part of the sequence overlapping with R1), and the downstream primer was attB2R2. The first round of PCR#1 amplification was performed using primers attB1F1/R1 to obtain the fragment containing the PVX CP gene-specific syn-tasiRNA sequence at the 3′ end. The second round of PCR#2 amplification was conducted using primers F2/attB2R2 to obtain the fragment containing the PVX CP gene-specific syn-tasiRNA sequence at the 5′ end. Subsequently, the full-length syn-tasiR-CPpvy/pvx3 fragment was obtained in the third round of PCR#3 amplification using the primers attB1F1/attB2R2 (Figure 3c). The overlap PCR3# fragment was ligated into the pMD19-T vector. After single-enzyme digestion of the vector, a BP reaction was performed to clone the fragment into the vector pDONR 207 and the expression vector pMDC32 using the Gateway recombination system. The final expression vectors were obtained and named as pMDC-syn-tasiR-CPpvy/pvx3 (syn-tasiR-CPpvy/pvx3) and pMDC-syn-tasiR-CPpvy/pvx5(syn-tasiR-CPpvy/pvx3). The expected specific amplified fragments with 239-bp long were amplified from *Agrobacterium* carrying pMDC-syn-tasiR-CPpvy/pvx3 and pMDC-syn-tasiR-CPpvy/pvx5 (Supplementary Figure S4). The expected fragments of 3236/627 and 3241/622 bp from pMDC-syn-tasiR-CPpvy/pvxs and pMDC-syn-tasiR-CPpvy/pvx5 were obtained by enzyme digestion with *PstI*, respectively (Figure 3d). Sequencing analysis revealed that both plasmids were correctly constructed (Figure S5).

### The transient expression of syn-tasiRNA-CPpvy/pvx and the antiviral effect targeting both PVY and PVX

To test the antiviral effects of syn-tasiRNA-CPpvy/pvx on both PVY and PVX, the expression vectors pMDC32-syn-tasiR-CPpvy/pvx and pMDC2-miR173 were transformed into *Agrobacterium* C58C1, respectively and co-infiltrated into the leaves of *N. benthamiana* (Figure 4a). At 3 days post infiltration, the extract mixture of PVYros and PVX was inoculated with manually infected leaves. Viral infection symptoms were observed at 14 dpi. The results showed that there were no viral infection symptoms of PVY and PVX in the six *N. benthamiana* plants transiently expressing syn-tasiR-CPpvy/pvx3 and syn-tasiR-CPpvy/pvx5, and distinct viral symptoms were observed on the leaves of the six *N. benthamiana* plants infiltrated with the empty vector pMDC32 (Figure 4b, c). Reverse transcription – quantitative PCR (RT-qPCR) analysis revealed that plants expressing syn-tasiR-CPpvy/pvx3 and syn-tasiR-CPpvy/pvx5 showed little to no viral accumulation. However, many PVY and PVX CP genes were detected in plants expressing the empty vectors (Figure 4d, e). This indicates that the transient expression of syn-tasiR-CPpvy/pvx3 and syn-tasiR-CPpvy/pvx5 can efficiently inhibit the infection of both viruses.

## Discussion

RNA silencing is a natural defense mechanism that has been engineered as a useful biotechnological tool for regulating plant gene expression.^1,46^ amiRNAs are valuable alternatives to RNA-silencing approaches based on the expression of large virus-derived sequences to generate antiviral resistance^14,^ .^47–49^ Based on syn-tasiRNAs, MIGS has been used as an RNA-silencing strategy to suppress gene expression and induce antiviral resistance.^29^

Syn-tasiR-FT targeting the flowering locus T (FT) gene and syn-tasiR-Trich targeting three MYB transcription factors (TRY, CPC, and ETC2) were transferred into *A. thaliana*, showing that syn-tasiRNAs can significantly delay plant flowering time and increase the number of trichomes in *A. thaliana* .^50^ Syn-tasiRNAs target the NbSu gene to induce NbSu gene silencing, and the silencing effect varies with the location of the syn-tasiRNAs.^51^ Syn-tasiRNA technology is advantageous for multiple aspects of gene silencing. First, it offers higher biological safety because syn-tasiRNAs are encoded by endogenous plant genes and are not prone to homologous recombination with endogenous genes. Second, it provides stronger interference by effectively improving the off-target effects of RNAi and enhancing its interference effects through the increased expression of multiple controllable 21-base tasiRNAs.^36^ Third, it offers the facility to acquire multiple types of resistance, as a single expression vector can simultaneously express multiple tasiRNAs to achieve the purpose of simultaneously interfering with multiple viruses. Finally, studies have shown that syn-tasiRNAs exhibit stable heritability. Syn-tasiRNAs can induce highly specific silencing throughout the plant, triggering local gene expression, regulating gene expression, and inducing resistance to viruses in the next generation of crops. This implies that gene silencing induced by syn-tasiRNA technology can retain stable traits after multiple generations.

In the present study, we used a previously reported TAS1/miR173-related system^29,30^ to express PVY-specific syn-tasiRNAs in *N. benthamiana*. The syn-tasiRNA constructs were designed to correspond to the CP-coding sequence of the PVY genome downstream of the miR173 cleavage site. In theory, tasiRNAs are thought to be derived from the DCL4-cleavage of RNA precursors synthesized by RDR6.^26,52^ In this case, the 21-nt syn-tasiRNAs were also processed by DCL4 in a phased manner following the miR173 cleavage site. However, the antiviral effects of the syn-tasiRNA constructs were not significant in the presence or absence of miR173.^22^

The leaves of *N. benthamiana* agroinfiltrated with syn-tasiR-CP2 and miR173 induced a strong sense of PTGS reaction with efficient anti-PVY protection. These plants transiently expressed syn-tasiR-CP3 but showed no anti-PVY effects. Viral accumulation in plants transiently expressing syn-tasiR-CP1 was significantly lower than in plants transiently expressing syn-tasiR-CP2. This indicated that the target sequence may be an important factor in the cleavage efficiency of syn-tasiRNAs for viral RNA. This is in agreement with a reported case in which the existence of construct-specific factors probably affected the processing and accumulation of particular syn-tasiRNAs.

A TAS1c-based construct expressing multiple distinct syn-tasiRNAs targeting the FT and Try genes triggered the silencing of multiple target transcripts and consequently blocked the corresponding plant phenotypes.^32^ Theoretically, these vectors can be designed to produce two or more syn-tasiRNAs to suppress additional unrelated targets. Syn-tasiRNA constructs targeting three turnip mosaic virus genes and three cucumber mosaic virus genes were created and introduced into *A. thaliana*, providing high resistance against the two viruses.^53^ This suggests the possibility of using a syn-tasiRNA strategy to resist three or more viral diseases. To determine whether a syn-tasiRNA vector carrying targets of both PVY and PVX could gain antiviral resistance against both viruses based on syn-tasiR-CPpvy2, we introduced the PVX target sequence to create syn-tasiR-CPpvy/pvx3 and syn-tasiR-CPpvy/pvx5. After the transient expression of the two constructs with pMDC-miR173 in *N. benthamiana*, the plants showed little or no viral infection. This indicates that syn-tasiRNAs targeting a single virus or two different viruses confer highly efficient antiviral resistance. Therefore, it is necessary to exploit their potential to target three or more molecules with high specificity.

Carbonell et al. showed that syn-tasiRNAs did not silence RNA more efficiently than did traditional amiRNAs.^32^ Additional attention should be paid to the advantages of syn-tasiRNAs, particularly with respect to the targeting of multiple viruses using a single construct.

## Materials and methods

### Plant materials and growth conditions

*N. benthamiana* plants were grown in a growth chamber at 22 ~ 26°C with a 16-h light/8-h dark photoperiod. Plants with four to five leaves, uniform growth, and size were selected for the experiments.

### Syn-tasiRnas design and synthesis

Syn-tasiRNAs can be processed by the expression of syn-tasiRNA transgenes containing functional TAS precursors, in which endogenous tasiRNA sequences are partially replaced by one or several concatenated syn-tasiRNA sequences.^54,55^ In this study, based on the sequence of the PVY CP gene (GenBank: KY780083.1), we searched for 126-bp long sequences, which were supposed to be processed into six syn-tasiRNAs (21 nt). Among them, “T” at the 5′ end in the second syn-tasiRNA and third syn-tasiRNA could be compulsory. Thus, three 126-bp conserved regions (176–302, 433–559, and 579–705 of the CP gene) were selected as the cleavage targets of syn-tasiRNAs. For each target gene, primers were designed by introducing the miR173 recognition site at the 5′ end (Table S1).

Total RNA was extracted from PVY-infected *N. benthamiana* plants using the TRizol reagent.^56^ cDNA was obtained using the Evo M-MLV plus 1^st^ strand cDNA synthesis kit (Accurate Biotechnology, China). Specific fragments of the PVY CP, which were supposed to be fused with the miR173 recognition site, were amplified by PCR. The corresponding PCR products of syn-tasiR-CPpvy1, syn-tasiR-CPpvy2, and syn-tasiR-CPpvy3 were analyzed by agarose gel electrophoresis. To create syn-tasiRNAs targeting both PVY and PVX in a single expression vector, primers were designed according to the syn-tasiR-CPpvy2 sequence and the selected target sequence of PVX (116–136 nt) (GenBank: M31541.1) (Table S2). Using syn-tasiR-CPpvy2 as the backbone, overlapping PCR was performed to introduce the target sequence of PVX CP at positions 3′D4(+) or 3′D6(+). The PCR products were detected by agarose gel electrophoresis and recovered using a standard agarose gel DNA recovery kit (BIOMIGA, China). The correctly sized PCR products were named syn-tasiR-CPpvy/pvx3 and syn-tasiR-CPpvy/pvx5, respectively.

### Plasmid construction

The PCR products of syn-tasiR-CPpvy1, syn-tasiR-CPpvy2, syn-tasiR-CPpvy3, syn-tasiR-CPpvy/pvx3, and syn-tasiR-CPpvy/pvx3 were cloned into the pMD19-T cloning vector, followed by PCR amplification and sequencing to verify successful integration and obtain pMD19-syn-tasiR-CPpvy1, pMD19-syn-tasiR-CPpvy2, pMD19-syn-tasiR-CPpvy3, pMD19-syn-tasiR-CPpvy/pvx3, and pMD19-syn-tasiR-CPpvy/pvx3. Using the BP reaction in the Gateway recombination system, these syn-tasiRNA bands were introduced into pDONR 207. LR reactions were then used to introduce these syn-tasiRNAs into the expression vector pMDC32 to develop pMDC-syn-tasiR-CPpvy1, pMDC-syn-tasiR-CPpvy2, pMDC-syn-tasiR-CPpvy3, pMDC-syn-tasiR-CPpvy/pvx3, and pMDC-syn-tasiR-CPpvy/pvx5. The plasmids were verified by enzymatic digestion or PCR amplification. The miR173 precursor sequence was amplified and cloned into the expression vector construct pMDC32 to obtain pMDC32-miR173, as described by Zhao et al., 2015.^22^

### Transient expression and virus infection assays

Transient expression assays in *N. benthamiana* leaves were performed as described by Zhao et al., 2015.^22^ The syn-tasiRNA constructs were transformed into the *Agrobacterium* strain C58C1 as described by Pasin et al., 2022^57^ *Agrobacterium* C58C1 carrying pMDC32-syn-tasiR-CPpvy1, pMDC32-syn-tasiR-CPpvy2, pMDC32-syn-tasiR-CPpvy3, pMDC32-syn-tasiR-CPpvy/pvx3, pMDC32-syn-tasiR-CPpvy/pvx5, and pMDC32-miR173 strains were incubated at 28°C for 16–18 h. The cultures were centrifuged at 5000 rpm at room temperature for 5 min. The supernatant was discarded and bacterial pellets were suspended at OD_600_ = 1 with a buffer containing 0.5 M2-(N-morpholino) ethanesulfonic acid hydrate (pH 5.6), 1 M MgCl_2_, and 0.1 M acetosyringone. The bacterial suspensions were incubated at room temperature (3 h) and then used to infiltrate the leaves of three-week-old *N. benthamiana* plants using a needleless syringe. The leaf samples infiltrated with the empty pMDC32 vector were used as controls. Each treatment included 12 or 6 plants and was repeated thrice. The treated *N. benthamiana* plants were grown in a greenhouse after infiltration.^58,59^ At 3 days post infiltration (dpi), the extracts of PVYros or PVX were inoculated onto the infiltrated leaves of each plant by mechanical inoculation, as described by Yue et al., 2022.^60^ The treated plants were grown in a greenhouse, and viral infection symptoms were observed at 12 or 18 dpi at different time points. The upper leaves of the plants showing obvious viral infection symptoms were taken and stocked in a freezer at − 80°C for subsequent analysis.

### Western blot analysis

Viral accumulation of PVY was determined by western blot analysis as described by Yue et al., 2023.^61^ Briefly, total protein extracts from the plant samples were prepared, resolved by SDS-PAGE, and electroblotted onto nitrocellulose membranes. The membranes were photographed as loading controls. Immunodetection was performed using rabbit anti-PVY CP antibody (AE012; ABclonal, China) as the primary antibody. Horseradish peroxidase-conjugated goat anti-rabbit IgG (ab205718; ABclonal, China) was used as the secondary antibody. Immunostained proteins were visualized by enhanced chemiluminescence detection (Clinx, China).

### RT-qPCR

Total RNA was purified and used in complementary DNA (cDNA) synthesis reactions, and cDNA aliquots were then used in PCR performed with the MonAmpTM SYBR Green qPCR mix (Monad, China) and gene-specific primers for PVY or PVX (Table S3) using an FTC-3000P Real-Time PCR system and Quantitative Thermal Cycler (Funglyn Biotech, Canada). Expression was normalized using NbUBI expression as a reference, and fold changes relative to the control value were calculated by the ΔΔCT method.^60^ Three independent biological replicates and three technical replicates for each biological sample were analyzed.

## Supplementary Material

Supplemental Material

## References

[1] Baulcombe D. RNA silencing. Trends in Biochemical Sciences. 2005;30(6):290–11. doi:10.1016/j.tibs.2005.04.012. 15950871

[2] Elbashir MS, Lendeckel W, Tuschl T. RNA interference is mediated by 21- and 22-nucleotide RNAs. Genes Dev. 2001;15(2):188–200. doi:10.1101/gad.862301. 11157775 PMC312613

[3] Fuchs U, Damm-Welk C, Borkhardt A. Silencing of disease-related genes by small interfering RNAs. Curr Mol Med. 2004;4(5):507–517. doi:10.2174/1566524043360492. 15267222

[4] Voinnet O. RNA silencing as a plant immune system against viruses[J]. Trends Genet. 2001;17(8):449–459. doi:10.1016/S0168-9525(01)02367-8. 11485817

[5] Ratcliff F, Harrison BD, Baulcombe DC. A similarity between viral defense and gene silencing in plants. Science. 1997;276(5318):1558–1560. doi:10.1126/science.276.5318.1558. 18610513

[6] Al-Kaff NS, Covey SN, Kreike MM, Page AM, Pinder R, Dale PJ. Transcriptional and posttranscriptional plant gene silencing in response to a pathogen. Science. 1998;279(5359):2113–2115. doi:10.1126/science.279.5359.2113. 9516113

[7] Paddison PJ, Caudy AA, Bernstein E, Hannon GJ, Conklin DS. Short hairpin RNAs (shRnas) induce sequence-specific silencing in mammalian cells. Genes Dev. 2002;16(8):948–958. doi:10.1101/gad.981002. 11959843 PMC152352

[8] Tenllado F, Diaz-Ruiz JR. Double-Stranded RNA-Mediated Interference with plant virus infection. J Virol. 2001;75(24):12288–12297. doi:10.1128/JVI.75.24.12288-12297.2001. 11711619 PMC116125

[9] Zhao MM, An DR, Zhao J, Huang GH, He ZH, Chen JY. Transiently expressed short hairpin RNA targeting 126 kda protein of tobacco mosaic virus interferes with virus infection. Acta Biochim Biophys Sin (Shanghai). 2006;38(1):22–28. doi:10.1111/j.1745-7270.2006.00124.x. 16395523

[10] Zhao MM, An DR, Huang GH, He ZH, Chen JY. A viral protein suppresses siRNA-directed interference in tobacco mosaic virus infection. Acta Biochim Biophys Sin (Shanghai). 2005;37(4):248–253. doi:10.1111/j.1745-7270.2005.00036.x. 15806291

[11] Simon-Mateo C, Garcia JA. MicroRNA-guided processing impairs plum pox virus replication, but the virus readily evolves to escape this silencing mechanism. J Virol. 2006;80(5):24–29. doi:10.1128/JVI.80.5.2429-2436.2006. PMC1395392 16474149

[12] Guo HS, Wang C-H, Fang R-X, Guo H-S. Artificial MicroRNAs highly accessible to targets confer efficient virus resistance in plants. J Virol. 2008;82(22):11084–11095. doi:10.1128/JVI.01377-08. 18768978 PMC2573272

[13] Niu QW, Lin SS, Reyes JL, Chen KC, Wu HW, Yeh SD, Nh C. Correction: Corrigendum: Expression of artificial microRnas in transgenic Arabidopsis thaliana confers virus resistance. Nat Biotechnol. 2007;25(2):254–254. doi:10.1038/nbt0207-254c. 17057702

[14] Qu J, Ye J, Fang R. Artificial MicroRNAs for Plant Virus Resistance. Methods in molecular biology (Clifton, N.J.). 2012;894:209–222. doi:10.1007/978-1-61779-882-5_14. 22678582

[15] Carbonell A, Carrington JC, Daròs JA. Fast-forward generation of effective artificial small RNAs for enhanced antiviral defense in plants. RNA & Disease. 2016;3(1):e1130. doi:10.14800/rd.1130. 26925463 PMC4768481

[16] Peragine A, Yoshikawa M, Wu G, Albrecht HL, Poethig RS. SGS3 and SGS2/SDE1/RDR6 are required for juvenile development and the production of trans-acting siRnas in Arabidopsis. Genes Dev. 2004;18(19):2368–2379. doi:10.1101/gad.1231804. 15466488 PMC522987

[17] Vazquez F, Vaucheret H, Rajagopalan R, Lepers C, Gasciolli V, Mallory AC, Hilbert JL, Bartel DP, Crété P. Endogenous trans-acting siRnas regulate the accumulation of Arabidopsis mRnas. Mol Cell. 2004;16(1):69–79. doi:10.1016/j.molcel.2004.09.028. 15469823

[18] Dunoyer P, Himber C, Voinnet O. DICER-LIKE 4 is required for RNA interference and produces the 21-nucleotide small interfering RNA component of the plant cell-to-cell silencing signal. Nat Genet. 2005;37(12):1356–1360. doi:10.1038/ng1675. 16273107

[19] Vaucheret H. MicroRNA-dependent trans-acting siRNA production. Sci STKE. 2005;2005(300):43. doi:10.1126/stke.3002005pe43. 16145017

[20] Talmor-Neiman M, Stav R, Klipcan L, Buxdorf K, Baulcombe DC, Arazi T. Identification of trans-acting siRnas in moss and an RNA-dependent RNA polymerase required for their biogenesis. Plant Journal. 2006;48(4):511–521. doi:10.1111/j.1365-313X.2006.02895.x. 17076803

[21] Gasciolli V, Mallory AC, Bartel DP, Vaucheret H. Partially redundant functions of Arabidopsis DICER-like enzymes and a role for DCL4 in producing trans-acting siRnas. Curr Biol. 2005;15(16):1494–1500. doi:10.1016/J.CUB.2005.07.024. 16040244

[22] Zhao M, San León D, Mesel F, García JA, Simón-Mateo C, Rao ALN. Assorted processing of synthetic trans-acting siRNAs and its activity in antiviral resistance. PLOS ONE. 2015;10(7):2–8. doi:10.1371/journal.pone.0132281. PMC4492489 26147769

[23] Fei Q, Xia R, Phased MB. Small interfering RNAs in posttranscriptional regulatory networks. Plant Cell. 2013;25(7):2400–2415. doi:10.1105/tpc.113.114652. 23881411 PMC3753373

[24] Axtell MJ, Jan C, Rajagopalan R, Bartel DP. A two-hit trigger for siRNA biogenesis in plants. Cell. 2006;127(3):565–577. doi:10.1016/j.cell.2006.09.032. 17081978

[25] Cuperus JT, Fahlgren N, Carrington JC. Evolution and Functional diversification of MIRNA genes. Plant Cell. 2011;23(2):431–442. doi:10.1105/tpc.110.082784. 21317375 PMC3077775

[26] Allen E, Xie Z, Gustafson AM, Carrington JC. microRNA-directed phasing during trans-acting siRNA biogenesis in plants. Cell. 2005;121(2):207–221. doi:10.1016/j.cell.2005.04.004. 15851028

[27] Yoshikawa M, Peragine A, Park MY, Poethig RS. A pathway for the biogenesis of trans -acting siRnas in Arabidopsis. Genes Dev. 2005;19(18):2164–2175. doi:10.1101/gad.1352605. 16131612 PMC1221887

[28] Gutiérrez-Nava MDLL, Aukerman MJ, Sakai H, Tingey SV, Williams RW. Artificial trans-acting siRnas confer consistent and effective gene silencing. Plant Physiol. 2008;47(2):543–551. doi:10.1104/pp.108.118307. PMC2409013 18441221

[29] Felippes FFD, Wang JW, Weigel D. MIGS: miRNA-induced gene silencing. Plant Journal. 2012;70(3):541–547. doi:10.1111/j.1365-313X.2011.04896.x. 22211571

[30] Montgomery TA, Yoo SJ, Fahlgren N, Gilbert SD, Howell MD, Sullivan CM, Alexander A, Nguyen G, Allen E, Ahn JH. et al. AGO1-miR173 complex initiates phased siRNA formation in plants. Proc Natl Acad Sci USA. 2008;105(51):20055–20062. doi:10.1073/pnas.0810241105. 19066226 PMC2598728

[31] Felippes FF, Weigel D. Triggering the formation of tasiRnas in Arabidopsis thaliana: the role of microRNA miR173. EMBO Rep. 2009;10(3):264–270. doi:10.1038/embor.2008.247. 19180117 PMC2658565

[32] Carbonell A, Takeda A, Fahlgren N, Johnson SC, Cuperus JT, Carrington JT. New generation of artificial MicroRNA and synthetic trans-acting small interfering RNA vectors for efficient gene silencing in Arabidopsis. Plant Physiol. 2014;165(1):15–29. doi:10.1104/pp.113.234989. 24647477 PMC4012576

[33] Singh A, Taneja J, Dasgupta I, Mukherjee SK. Development of plants resistant to tomato geminiviruses using artificial trans-acting small interfering RNA. Mol Plant Pathol. 2015;16(7):724–734. doi:10.1111/mpp.12229. 25512230 PMC6638473

[34] Baykal U, Liu H, Chen X, Nguyen HT, Zhang ZJ. Novel constructs for efficient cloning of sRNA-encoding DNA and uniform silencing of plant genes employing artificial trans-acting small interfering RNA. Plant Cell Rep. 2016;35(10):2137–2150. doi:10.1007/s00299-016-2024-9. 27417696

[35] Carbonell A, Daròs JA. Artificial microRnas and synthetic trans-acting small interfering RNAs interfere with viroid infection. Mol Plant Pathol. 2017;18(5):746–753. doi:10.1111/mpp.12529. 28026103 PMC6638287

[36] Carbonell A, Lisón P, Daròs JA. Multi-targeting of viral RNAs with synthetic trans-acting small interfering RNAs enhances plant antiviral resistance. Plant Journal. 2019 Nov. 100(4):720–737. doi:10.1111/tpj.14466. PMC6899541 31350772

[37] Wu B, Jiang SS, Zhang M, Xu KD, Xin ZM, Wang SJ. Constructing Transgenic Tobacco with Resistance against Potato Virus Y Using Synthetic Trans—siRNA. Shandong Agri Sci. 2018;50(12):86–90. doi:10.14083/j.issn.1001-4942.2018.12.017.

[38] Miao S, Liang C, Li J, Baker B, Luo L. Polycistronic artificial microRNA-mediated resistance to cucumber green mottle mosaic virus in cucumber. Int J Mol Sci. 2021;22(22):12237. doi:10.3390/ijms222212237. 34830122 PMC8620374

[39] Vreugdenhil D, Bradshaw J, Gebhardt C, Govers F, Ross HA. Potato biology and biotechnology: advances and perspectives[M]. Amsterdam The Netherlands Elsevier B. 2007; doi:10.1016/B978-0-444-51018-1.X5040-4.

[40] Revers F, García JA. Molecular biology of potyviruses. Adv Virus Res. 2015;92:101–199. doi:10.1016/bs.aivir.2014.11.006. 25701887

[41] Yang X, Li Y, Wang A. Wang A. Research advances in potyviruses: From the laboratory bench to the field. Annu Rev Phytopathol. 2021;59(1):1–29. doi:10.1146/annurev-phyto-020620-114550. 33891829

[42] Rajamäki ML, Kelloniemi J, Alminaite A, Kekarainen T, Rabenstein F, Valkonen JP. A novel insertion site inside the potyvirus P1 cistron allows expression of heterologous proteins and suggests some P1 functions. Virology. 2005;342(1):88–101. doi:10.1016/j.virol.2005.07.019. 16112702

[43] Bance VB. Replication of potato virus X RNA is altered in coinfections with potato virus Y. Virology. 1991;182(2):486–494. doi:10.1016/0042-6822(91)90589-4. 2024486

[44] Dickison L, Singh XX, Nie XZ, Xiong X, Nie X. Studies of tomato plants in response to infections with PVX and different PVY isolates reveal a remarkable PVX-PVYNTN synergism and diverse expression profiles of genes involved in different pathways. Eur J Plant Pathol. 2016;144(1):55–71. doi:10.1007/s10658-015-0750-4.

[45] Huisman MJ, Linthorst HJ, Bol JF, Cornelissen JC. The complete nucleotide sequence of potato virus X and its homologies at the amino acid level with various plus-stranded RNA viruses. J Gen Virol. 1988;69(8):1789–1798. doi:10.1099/0022-1317-69-8-1789. 3404114

[46] Simón-Mateo C, García JA. Antiviral strategies in plants based on RNA silencing. Biochim Biophys Acta. 2011;1809(11–12):722–731. doi:10.1016/j.bbagrm.2011.05.011. 21652000

[47] García JA, Simón-Mateo C. A micropunch against plant viruses. Nat Biotechnol. 2006;24(11):1358–1359. doi:10.1038/nbt1106-1358. 17093480

[48] Tiwari M, Sharma D, Trivedi PK. Artificial microRNA mediated gene silencing in plants: progress and perspectives. Plant Mol Biol. 2014;86(1–2):1–18. doi:10.1007/s11103-014-0224-7. 25022825

[49] Mesel F, Zhao M, García B, Simón-Mateo C, García JA. Targeting of genomic and negative-sense strands of viral RNA contributes to antiviral resistance mediated by artificial miRnas and promotes the emergence of complex viral populations. Mol Plant Pathol. 2022;23(11):1640–1657. doi:10.1111/mpp.13258. 35989243 PMC9562735

[50] López-Dolz L, Spada M, Daròs JA, Carbonell A. Fine-tune control of targeted RNAi efficacy by plant artificial small RNAs. Nucleic Acids Res. 2020;48(11):6234–6250. doi:10.1093/nar/gkaa343. 32396204 PMC7293048

[51] Cisneros AE, Torre-Montaña ADL, Carbonell A. Systemic silencing of an endogenous plant gene by two classes of mobile 21-nucleotide artificial small RNAs. Plant Journal. 2022;110(4):1166–1181. doi:10.1111/tpj.15730. PMC9310713 35277899

[52] Howell MD, Fahlgren N, Chapman EJ, Cumbie JS, Sullivan CM, Givan SA, Kasschau KD, Carrington JC. Genome-wide analysis of the RNA-DEPENDENT RNA POLYMERASE6/DICER-LIKE4 pathway in Arabidopsis reveals dependency on miRNA- and tasiRNA-directed targeting. The Plant Cell Online. 2007;19(3):926–942. doi:10.1105/tpc.107.050062. PMC1867363 17400893

[53] Chen L, Cheng X, Cai J, Zhan L, Wu X, Liu Q, Wu X. Multiple virus resistance using artificial trans-acting siRnas. J Virol Methods. 2016;228:16–20. doi:10.1016/j.jviromet.2015.11.004. 26562057

[54] Bohnsack MT, Czaplinski K, Gorlich D. Exportin 5 is a RanGTP-dependent dsRNA-binding protein that mediates nuclear export of pre-miRnas. RNA-a publication of the RNA society. 2004;10(2):185–191. doi:10.1261/rna.5167604. PMC1370530 14730017

[55] Axtell MJ. Classification and comparison of small RNAs from plants. Annu Rev Plant Biol. 2013;64(1):137–159. doi:10.1146/annurev-arplant-050312-120043. 23330790

[56] Cuperus JT, Carbonell A, Fahlgren N, Garcia-Ruiz H, Burke RT, Takeda A, Sullivan CM, Gilbert SD, Montgomery TA, Carrington JC. Unique functionality of 22-nt miRnas in triggering RDR6-dependent siRNA biogenesis from target transcripts in Arabidopsis. Nature Structural & Molecular Biology. 2010;17(8):997–1003. doi:10.1038/nsmb.1866. PMC2916640 20562854

[57] Pasin F. Assembly of plant virus agroinfectious clones using biological material or DNA synthesis. STAR Protoc. 2022;3(4):101716. doi:10.1016/j.xpro.2022.101716. 36149792 PMC9519601

[58] Carbonell A, Daròs JA. Design, synthesis, and functional analysis of highly specific artificial small RNAs with antiviral activity in plants. Antiviral Plant Artificial Small RNAs. 2019;2028:231–246. doi:10.1007/978-1-4939-9635-3_13. 31228118

[59] Carbonell A. Design and high-throughput generation of artificial small RNA constructs for plants. Methods Mol Biol. 2019;1932:247–260. doi:10.1007/978-1-4939-9042-9_19. 30701506

[60] Yue JY, Wei Y, Sun ZQ, Chen YH, Wei XF, Wang HJ, Pasin F, Zhao MM. AlkB RNA demethylase homologues and N6-methyladenosine are involved in potyvirus infection. Mol Plant Pathol. 2022;23(10):1555–1564. doi:10.1111/mpp.13239. 35700092 PMC9452765

[61] Yue JY, Lu Y, Sun ZQ, Guo YQ, San León D, Pasin F, Zhao MM. Methyltransferase-like (METTL) homologues participate in nicotiana benthamiana antiviral responses. Plant Signal Behav. 2023;18(1):2214760. doi:10.1080/15592324.2023.2214760. 37210738 PMC10202045

