# Supplementary material for: syn-tasiRnas targeting the coat protein of potato virus Y confer antiviral resistance in Nicotiana benthamiana
Source: Plant Signal Behav. 2024 May 26;19(1):2358270. doi: 10.1080/15592324.2024.2358270 (PMC11135832; doi:10.1080/15592324.2024.2358270)
Supplement: Supplemental Material [file KPSB_A_2358270_SM2219.zip › Table S1-3.docx]

**Table S1 Primers used for PCR**

| Name of primers | Sequence of primer | Size of the sequence (nt) | Size of expected fragments (bp) |
| --- | --- | --- | --- |
| tasiR-CPpvy1-F | GGGGACAAGTTTGTACAAAAAAGCAGGCTTTGTGATTTTTCTCTACAAGCGAAATGTCGTATGCAAGTTGTACT | 74  50 | 207 |
| tasiR-CPpvy1-R | GGGGACCACTTTGTACAAGAAAGCTGGGTTGCAACTGT ACTAAATTTGGA |  |  |
| tasiR-CPpvy2-F | GGGGACAAGTTTGTACAAAAAAGCAGGCTTTGTGATTTTTCTCTACAAGCGAATTACGAACTAAACCATATCGT | 74  50 | 207 |
| tasiR-CPpvy2-R | GGGGACCACTTTGTACAAGAAAGCTGGGTAATCGTTGA GAATGCAAAACC |  |  |
| tasiR-CPpvy3-F | GGGGACAAGTTTGTACAAAAAAGCAGGCTTTGTGATTTTTCTCTACAAGCGAAACCATCCAATCCGAA AAGTCG | 74  50 | 207 |
| tasiR-CPpvy3-R | GGGGACCACTTTGTACAAGAAAGCTGGGTGCTCGCTATGCTTTTGACTTT |  |  |

**Table S2 Primers used for Overlap PCR**

| Name of primers | Sequence of primer | Size of the primers (nt) | Size of expected fragments (bp) |
| --- | --- | --- | --- |
| tasiR-CPpvy/pvx3-F | TGTCTGAGGATCAACTTCCTTTGAGAAATGTGCCATGATTTG | 42 | 239 |
| tasiR-CPpvy/pvx3-R | AAGGAAGTTGATCCTCAGACAGAAATGCGCAACAAAAAGGAACC | 44 |  |
| tasiR-CPpvy/pvx5-F | TGTCTGAGGATCAACTTCCTTCTCAACGATTACCC | 36 | 239 |
| tasiR-CPpvy/pvx5-R | AAGGAAGTTGATCCTCAGACACAAATCATGGCACATTTCTCAG | 43 |  |

**Table S3 Primers used for RT-qPCR**

| Name of primers | Sequence of primer | Size of the sequence (nt) | Size of expected fragments(bp) |
| --- | --- | --- | --- |
| q-PVYCP-F | ACACCAGTGAGGGCTAGGGA | 21 | 170 |
| q-PVYCP-R | CATCCTCGGTGGTGTGCCTC | 21 |  |
| q-PVXCP-F | GACATGAAGGTGCCCACAGA | 20 | 134 |
| q-PVXCP-R | CTAGCTCTGCTGATGCCGTT | 20 |  |
