# Supplementary figures and images for: syn-tasiRnas targeting the coat protein of potato virus Y confer antiviral resistance in Nicotiana benthamiana
Source: Plant Signal Behav. 2024 May 26;19(1):2358270. doi: 10.1080/15592324.2024.2358270 (PMC11135832; doi:10.1080/15592324.2024.2358270)

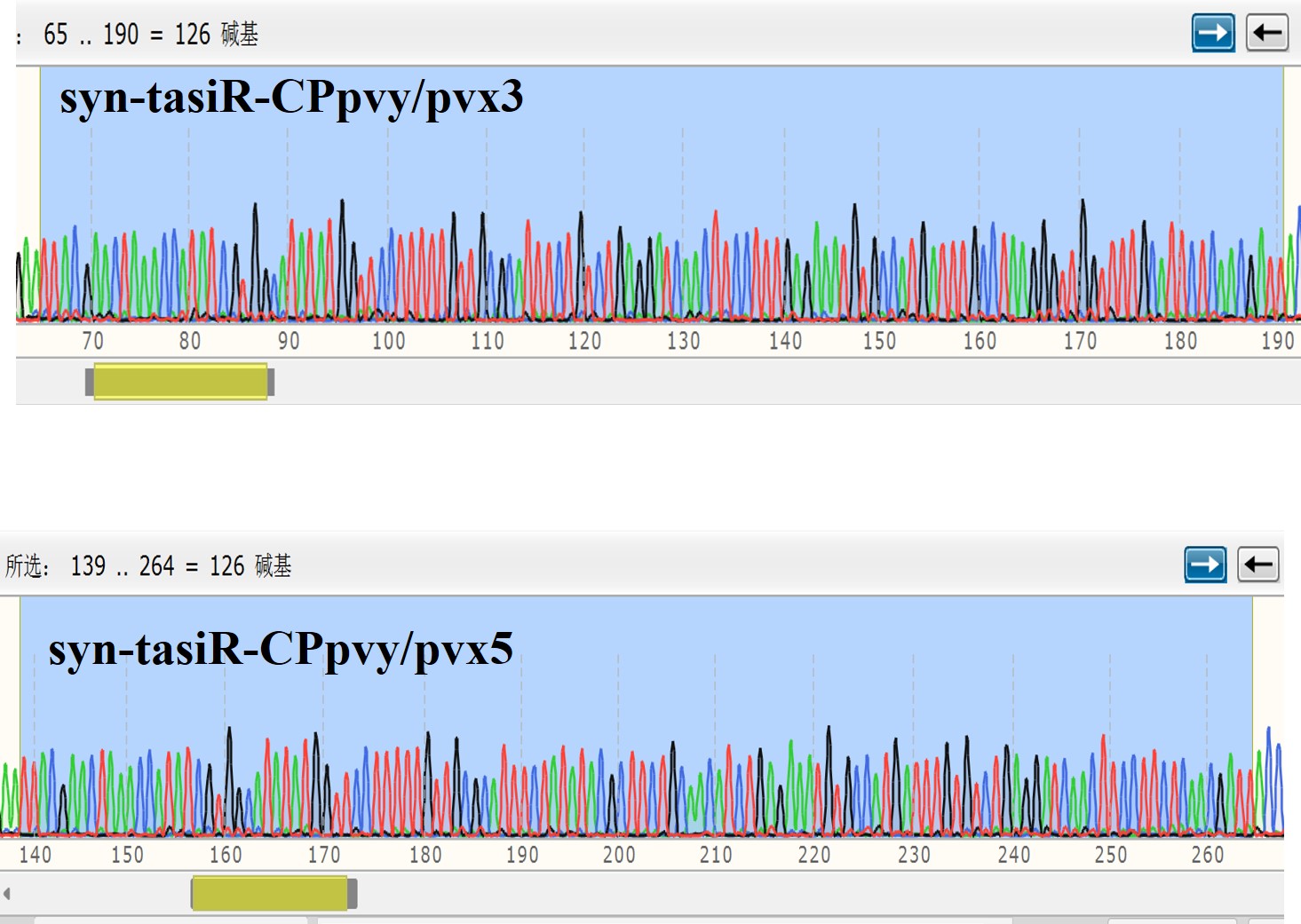

Supplement: Supplemental Material [file KPSB_A_2358270_SM2219.zip › Fig S1.jpg]

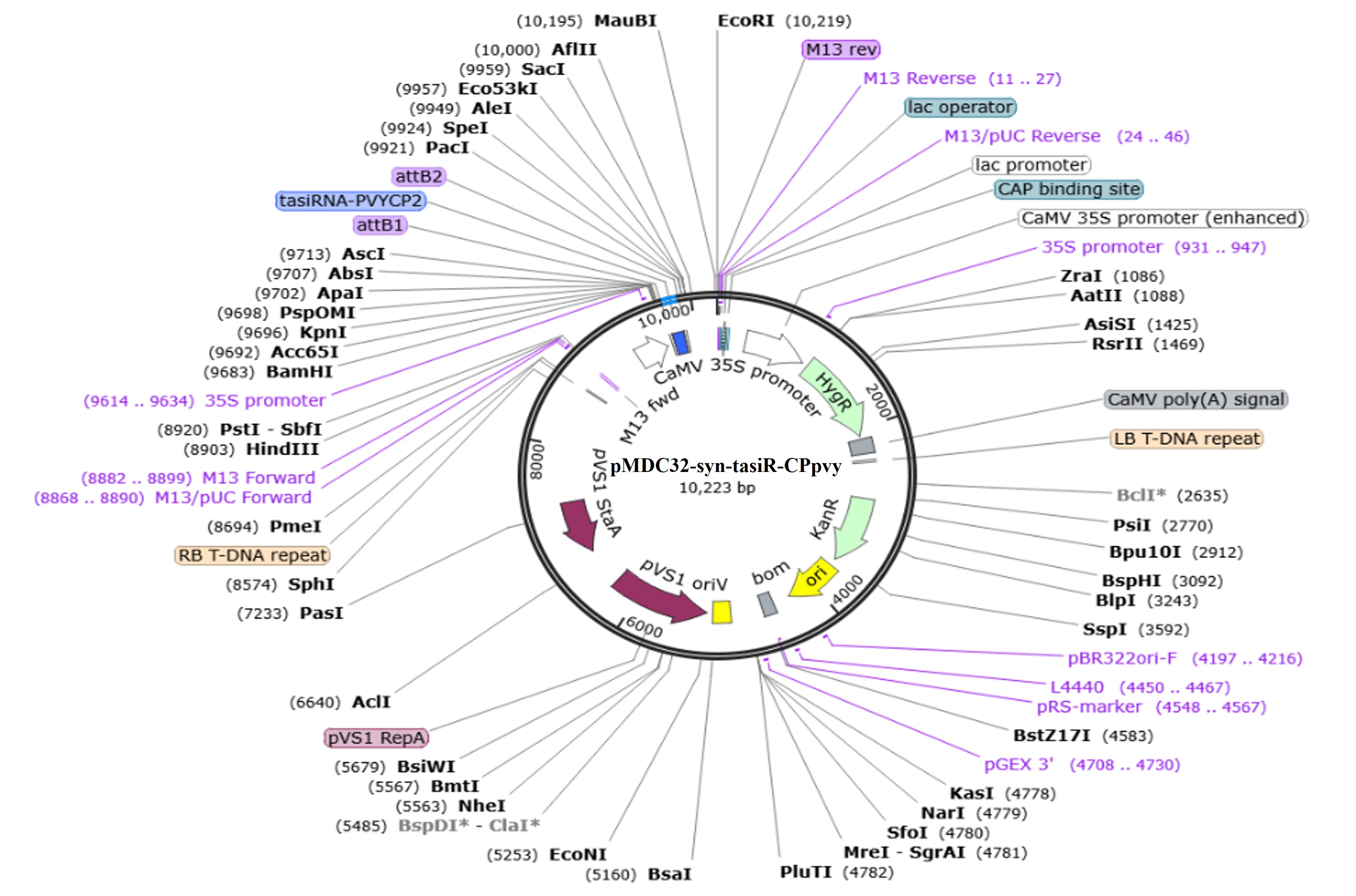

Supplement: Supplemental Material [file KPSB_A_2358270_SM2219.zip › Fig S2.jpg]

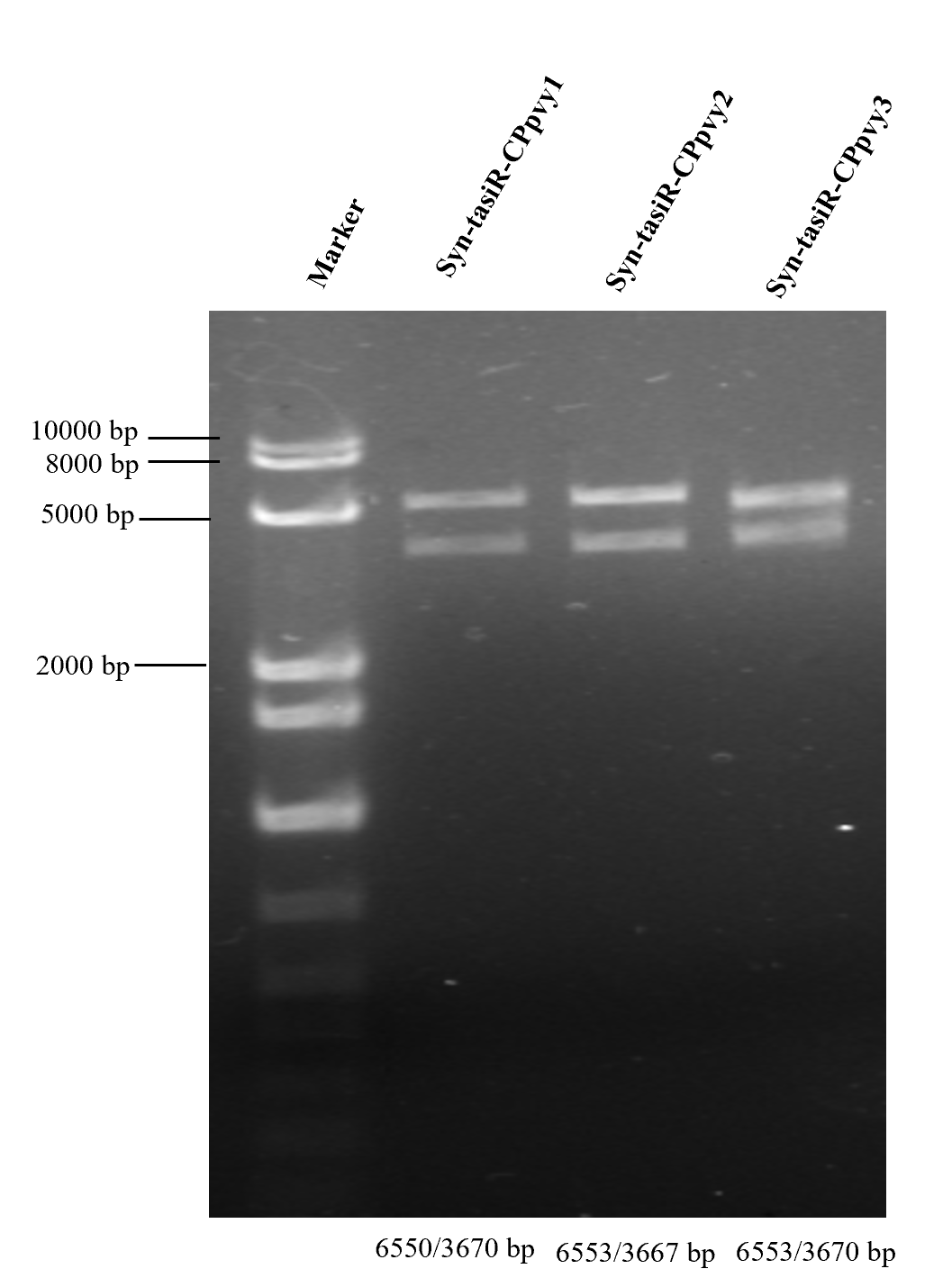

Supplement: Supplemental Material [file KPSB_A_2358270_SM2219.zip › Fig S3.png]

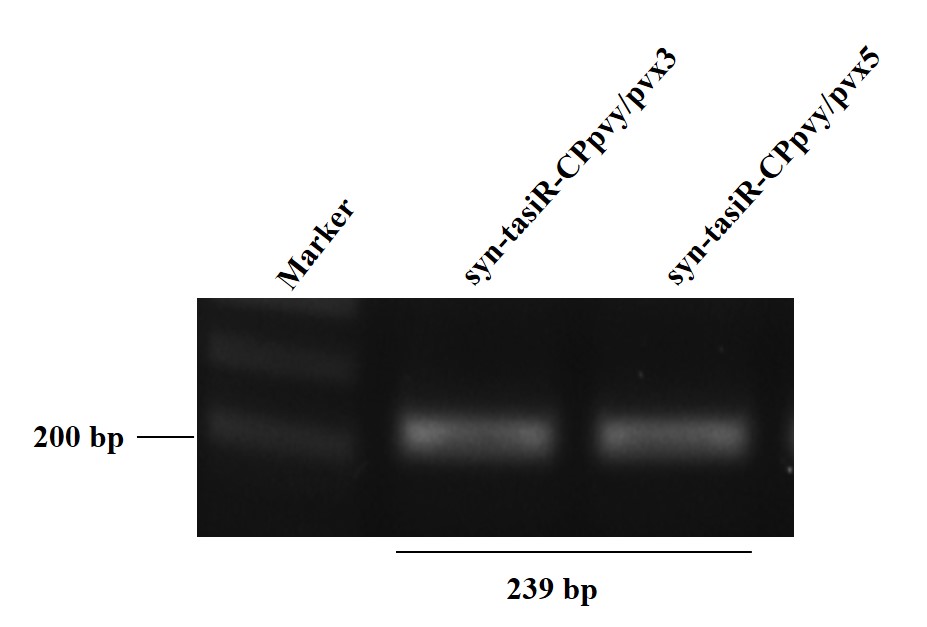

Supplement: Supplemental Material [file KPSB_A_2358270_SM2219.zip › Fig S4.jpg]

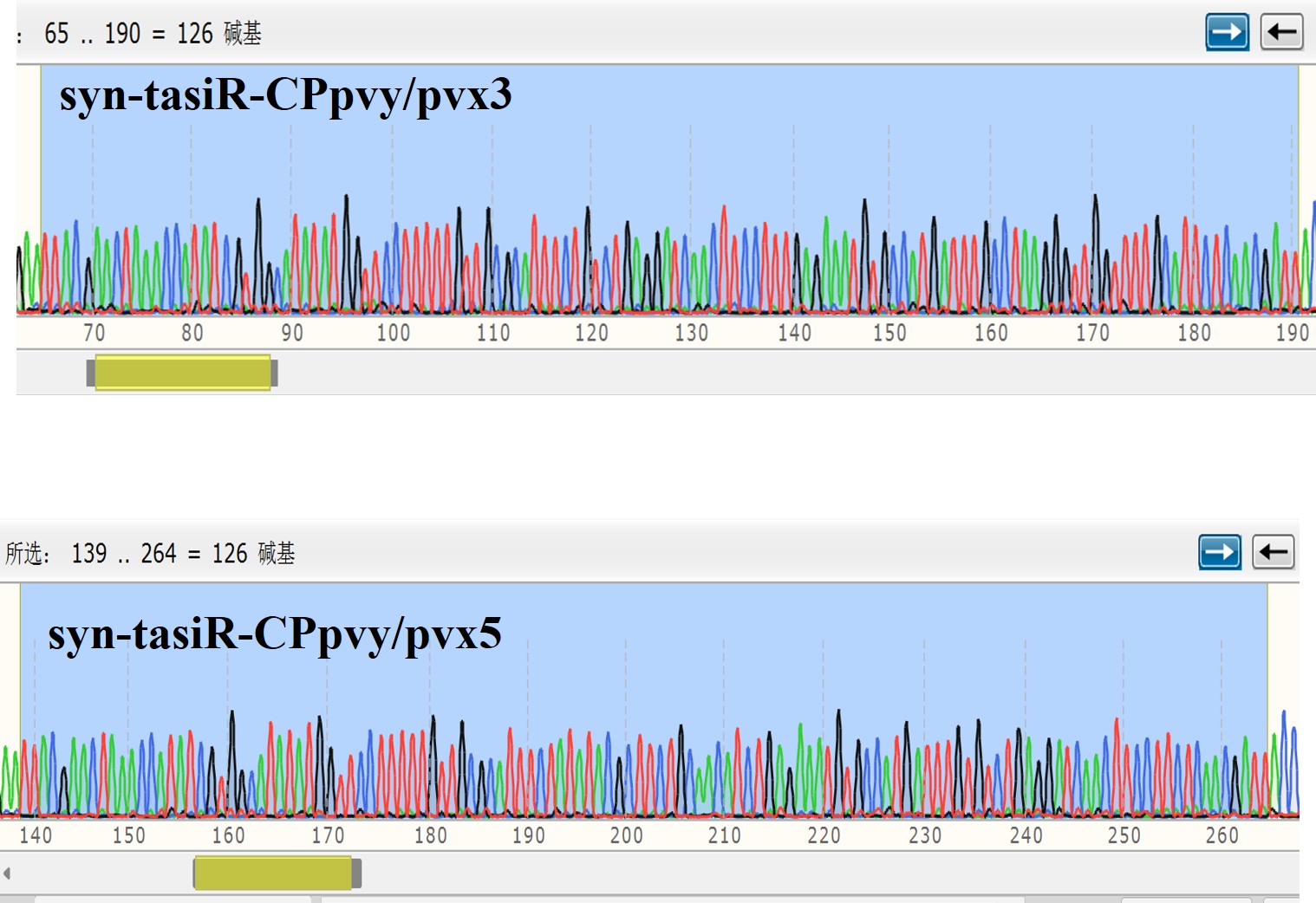

Supplement: Supplemental Material [file KPSB_A_2358270_SM2219.zip › Fig S5.jpg]

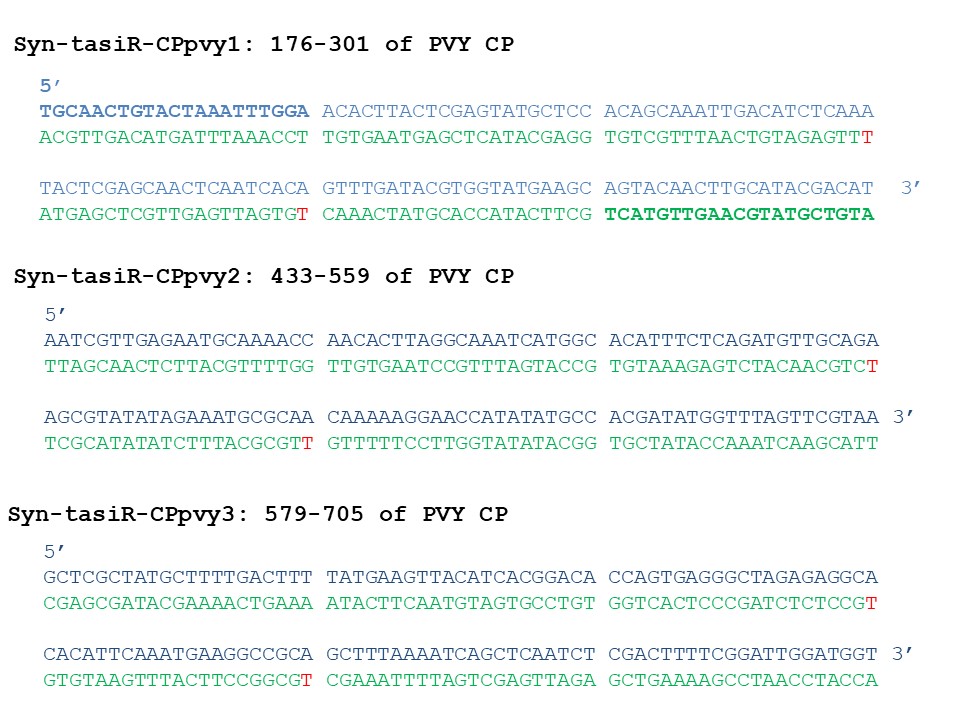

Supplement: Supplemental Material [file KPSB_A_2358270_SM2219.zip › Fig S6.jpg]
